# Organization of the American Medical Association

**Published:** 1907-08

**Authors:** 


### ORGANIZATION OF THE AMERICAN MEDICAL ASSOCIATION.

So excellent and timely is the following editorial from the May issue of American Medicine that we quote it in full in spite of its length.

THE DEFECTS IN THE PLAN OF ORGANIZATION OF THE AMERICAN MEDICAL ASSOCIATION are few, but are becoming apparent through the experiences of five years trial. They must be remedied, and soon, by those within the organization or they will be seized upon by its enemies and those of the profession. When criticism is rife it is short-sighted and foolish to think that betterment is impossible. It is a sorry blunder to oppose discussion of weakness, to claim infallibility in conception or execution. If plasticity is lost growth and progress are stopped. Criticism within the ranks is usually by minorities, progress is initiated by the few. The movement for the unification of the profession had its rise with a very few men, of constructive statesmanship who worked for the establishment of an *esprit de corps,* the raising of professional standards, the increase of scientific work, and through these the advancement of the higher hemanities. The profession would benefit by demonstration of its solidarity and by protection against exploitation for commercial or political advantage.

THE EVIL OF TOO MUCH OFFICIALISM are recognized generally throughout the profession, and protest is common. It can be given no clearer utterance than in the recent address of President-elect Bryant before the united medical societies of the State of New York. Note the ringing words concerning the dangers of paternalism: "It would be sadly amiss, indeed, were I not to admonish you at the opening of a united career, of the grave perils by the spirit of paternalism which, not infrequently, is a pernicious by-product of organized power. The seductive and oppressive influence of this spirit often usurps, and may inhibit that nobler, more enduring and beneficent one, characteristic of common manhood and of professional brotherhood. In this relation it should not be forgotten that the greater and more extended is the membership of a fraternal body, the greater and more extended its constituent paternal desires are apt to be. Consequently, the longer the chain of affiliation is, the weaker it may become, for, as you already understand, no chain is stronger than its weakest link."

CENTRALIZATION OF POWER MEANS OPPORTUNITY FOR CORRUPTION by concentration of temptation. It is alleged that the American Medical Association in its present form is simply an elected autocracy instead of the republican institution planned. Office held too long and without proper restriction may come to be regarded as a vested right. Large bodies are proverbially inert and individuals are prone to seize advantage. Criticism thus far is largely confined to the existence of possibilities. There seems yet no belief in much wrong doing, nor widespread distrust of individuals. Yet thoughtful men are beginning to recognise the possibility of abuse because too much is trusted to the altruism and honesty of officials and socalled leaders. Too often the politician benefits chiefly or alone by what the statesman has built up. When power is vested in the hands of the few, who may use official positions for private advantage, corruption and graft are more than possibilities. In any governing or legislative body honesty and identity of interest will inspire true representation. But when that body is uncontrolled save by periodic elections, and is without direct recourse to popular endorsement or reversal of particular acts, the representtatives are free to legislate in their own interests collectively or individually, or in the interests of others who furnish sufficient inducements. The duty and the self-interest of functionaries do not always agree, and self-interest has many disguises. In office, when possibility of revision of their acts is negligible, good men are difficult to keep good.

THE PROVISION OF THE REFERENDUM in the constitution of the American Medical Association was intended to protect the members against these possibilities, but in its present form it is inadequate. It is optional instead of obligatory; it is legislative only, there being no provision for reference to popular vote save upon the initiative of one or the other general body of representatives; the proportion of votes required to obtain the referendum is too great; no provision is made for general secret vote on the part of the members of the Association. Instead of self-government, the members of the Association possess only the privilege of electing their governors —the delegates—and through them the trustees. If wrongly represented by any official act there is no recourse other than to elect other governors at certain specified times. True, these delegates and trustees are supposed to be men of self-sacrificing spirit, who serve without recompense or personal advantage. Yet there seem to be evidences that the fine Italian hand of the politician has been at work. Why has work for self-election as delegate been so industriously prosecuted in more than one section and State society? A certain trustee, whose special work has seemed to lie in directing policies, financial and otherwise, of the Association, is said to have spent in visiting delegates much of the three months preceding the meeting at which his reelection or the election of his successor was due—"mending fences" is the political phrase. Perhaps his action was ruled by altruism, not love of power. But the power nevertheless is his for the full term of his election.

THE RIGHTS OF INDIVIDUALS IN PROFESSIONAL ORGANIZATIONS are strongly upheld by the vast majority of the right-thinking men of the profession. Their beliefs are ably voiced by President Bryant, who thus warns and advises his professional brethren: "Kindly permit me to admonish you to be steadfast and diligent in the preservation and development of the State organization as at preesnt constituted. It is a free government in the complete sense of the expression, one in which the humblest members of the most cheerless stations of the State have equal privileges with those of exalted station in the midst of wealth and power; one in which the officers are your servants and not your masters, unless you so will it to be, and from whom you have the right to demand at the proper time, and in a suitable manner, a complete accounting of the status of their respective stewardships, and which, when wisely required, they have no right to decline. And in all other respects, your rights and privileges cannot be overshadowed by those of another unless you, yourselves, lend aid to the eclipse."

THE ESTABLISHMENT OF THE PRINCIPLE OF SELF-GOVERNMENT IS NECESSARY.—In spite of the natural conservatism of medical men, there is a growing discontent, a feeling that the real sentiment of the profession is not truly represented in the increasing tendency of the "leaders" toward mere money-getting, monopoly, and trade-unionism. This discontent is not reactionary but constructive. It demands the retention of all the advantages obtained through the late reorganization and the adding of others to make these more effective and more truly representative. President Bryant expressed this feeling of the profession admirably when he recently said: "If on our part, a spirit of fraternal oppression of any kind should develop as the outcome of organized strength, then indeed will the day of consolidation become one of mourning for the loss in fraternal fellowship and in professional station, instead as it ought, a day of rejoicing because of the great opportunity for general good thus brought into existence."

IMPORTANT MATTERS OF GENERAL POLICY SHOULD BE REFERRED TO THE BODY OF MEMBERS OF THE ASSOCIATION.—The vast equipment of the Journal and the Association can be economically used to this end, each member being afforded opportunity through his district or county society to record a secret ballot giving his judgment. A petition signed by 500 mmbers of the Association or five per cent of either the House of Delegates or the General Session should ensure reference to the general ballot. Save in emergency cases, this may be deferred to times when several matters can be acted upon together, or at certain fixed periods of the year. The spirit of fair-play which animates the profession as a whole may be trusted to prevent injustice and reach a general rightness. No better curb upon extravagance of either power or expenditure can be conceived. There is danger, under the present organization, of the plenary powers of the trustees being regarded as proprietary rights. These nine men (with the President and Secretary) may divert portions of the vast accumulations possible under the present policies to ends not in accord with the original aims of the Association nor thoroughly representative of the great body of the profession. One may instance such steps as entering into competition with publishers of books or with the manufacturers of drugs, in the name of the Association and by use of its funds. The real aims in such measures may be masked by a specious plea for purity and professionalism. Even the admirable Council of Pharmacy might be made to further such schemes by clearing the field of competitors.

THE JOURNAL IS THE PROPERTY OF THE MEMBERS OF THE ASSOCIATION, not of its trustees or officers. It must, therefore, be conducted in the spirit of the profession at its best, not as may seem good to any individual or coterie. Much of the danger of misrepresentation of the will of the general body of the Association and of the profession is particularly possible with regard to the official organ of the Association. There should be the utmost freedom of discussion in the pages of the Journal of all matters relating in any way to the interests of the profession. This should apply especially to the proceedings of the House of Delegates, which should be published verbatim. The most obscure member of the Association, who cannot afford to leave practice and spend money to be present at the deliberations of his representative body, is entitled to know not only what scientific work has been done at the annual meeting, but also in what manner its financial affairs are conducted, what affairs come before its legislative sessions, the matter and manner of debate, and the votes recorded on any subject.

EVERY CONSTITUENCY IS ENTITLED TO KNOW HOW ITS REPRESENTATIVE REPRESENTS.—Concerning this contention President Bryant has nobly said: "Since 'every rose has its thorn,' it should not be overlooked at this time that the flowery probabilities of mutual scientific medical journalism might easily be inhibited, and even destroyed by the thorny possibilities of selfish time-serving desires. In any event, common justice demands that all questions of mutual, material interests, and others of general interests to the profession of the State, should find free and impartial utterance through the pages of its own journal. The presence of a spirit which contemplates that the entire membership of the profession of this State be not made familiar by its own servants with the facts relating to the respective differences in all matters of common importance, is offensive to the principles of American institutions, and to those of fair dealing in all lines of manly action." . . . "The fact that each member of the organized body has rights in the publication equal to those of another, would seem to inspire sentiments of mutual interest and fidelity, resulting in a more extended and higher standard of professional fellowship, than sometimes appears to exist. And, too, the united voice of the profession can be heard through the agency of the Journal, with a clear unhindered emphasis on all matters relating to common professional and common public betterment."

THE POWERS AND FUNCTIONS OF CERTAIN OFFICERS ARE TOO GREAT.—This applies not only to elected officers, but to those appointed by the trustees. Of the five lucrative offices at the disposal of the Association, four are assigned not by the members of the Association, nor even by their representatives in the House of Delegates, but by the trustees. And these functionaries are directly accountable to the trustees alone. Two of these officials, the Organizer and the Advertising Manager, each hold but one office. The other two, General Business Manager and Editor, the most important, are represented by one individual, who also combines with these appointive offices the elective position of General Secretary of the Association. From the standpoint of power and opportunity, the secretaryship may be made far more important than the presidency, which can be held for one year only. In times past presidential communications have been received with the proper residential date line but bearing a Chicago post-mark several days later. This would seem to show that certain Presidents may acknowledge the supremacy of the Secretary or the Editor! By a curious oversight the Secretary, the Editor, and the General Business Manager each forms a strong factor in the bodies governing his actions, compensation, policy, etc. It is stated that the incumbent of these offices is present at all the meetings in an attitude of practically determining all decisions. When the Association had only 10,000 members this concentration of office seemed a measure of economy both of money and effort. The Association has outgrown any possible benefits of such Each of these offices must be filled by some man particularly fitted to discharge its duties, or worse will come. The Association has sufficient wealth at its command to obtain the proper men and to compensate them worthily. No one man can by any gifts of nature or acquirement properly perform these diverse duties. That the present able Business Manager has so long filled the two other important offices is far from creditable to his employers. He should be continued as Business Manager at a liberal salary; the Association might even afford to pay for the one office the sum this functionary has been receiving for the combination position.

THE DUTIES AND FUNCTIONS OF THE EDITOR OF THE JOURNAL MUST BE MORE CLEARLY DEFINED.—He cannot be, in any sense, "independent," nor can he have discretionary power. The Journal must mirror the sentiment of the Association as a whole, but must give due representation to minorities. The unknown and presumably unimportant member is as much the owner of the Journal as any elected or appointed functionary. It is impossible to "divorce the Journal and the Association." The strictest justice and impartiality must be the only policy of both. Personal judgment and the gratifying of private piques are indefensible. To this end, although the Editor should have absolute freedom of literary revision, the right of determining the scientific and other contents of the Journal must rest with the sections and the Association. These requirements emphasize the necessity of high literary and professional attainments for the Editor of the Journal. He must be scholarly. He must be able to assist contributors to clothe their contributions so that through its Journal the work of the Association may be properly presented. He must be identified with the highest professional standards, the most progressive thought, and must have the judiciary faculty of impartiality. The Editor is not required to give ex parte judgements upon either matters of science or ethics, nor to instruct in matters of duty. No member of the Association should be humiliated by refusal of admission to the pages of the Journal by the judgment of one man or several as to the scientific value of his communication. Nor should he, by one man's decision, or that of a small committee, be advised to withdraw "for his own good" or because his "motives are open to suspicion." Courtesy and consideration without arrogance must rule in all editorial communications; at least until omniscience is really an editorial attribute.

THE SECRETARY OF THE ASSOCIATION represents the Association most peculiarly, not alone to individual members, but also among the scientific bodies of the world. He should be a gentleman in the best sense of the word, fitted by professional and general training and education to meet the highest and the most humble worthily. He should possess an agreeable and magnetic personality, and be imbued with professional ideals. He must be able to inspire respect for the Association he represents because he represents it with dignity and honor. If any two offices in the gift of the Association can be fitly combined, those of Organizer and Secretary are the two. The office of Organizer has been regarded as temporary, but, if the Association is to continue to grow, many of the duties of the office will remain permanently. The two offices require the same type of man. Their duties are sufficiently similar to enable a profitable consolidation. There should be regular itemized reports of the constructive work done by this Secretary-Organizer. This will enable the Association to judge of the possibility of futhrer development and adjustment. This office, as well as that of Editor, should be well paid in order that the best talent may be obtained. The Association can afford to appropriate at least $25,000 to divide in salaries among its Editor, Secretary-Organizer, and Business Manager. The increase over the salaries now paid would not be exorbitant, and would be a small price to pay for the increase in efficiency and the better standing of the Association among similar bodies.

CONSTITUENT OWNERSHIP SHOULD BE THE KEYNOTE OF BOTH THE ASSOCIATION AND ITS JOURNAL.— That the Journal of the Association no longer represents the policy of the Association save so far as its policy and that of certain officials coincide is well known. Representation in the Journal has been denied time and again to those who venture to disagree with the sentiments and policies of those who constitute themselves the court of last appeal. It is stated that threats even have been used that the whole power of the Association Machine would be brought to bear against a recalcitrant, to insure his humiliation and disgrace, unless he would forego the publication of his side of the case. Power to exclude a fellow-member of the Association from any of its rights and privileges must be carefully withheld from any functionary or set of officials. It was with reference to such possibility that President Bryant recently said: "The editorial policy of a journal of constituent ownership, like this of ours, should be guided by the composite wisdom of an impartial committee chosen for the purpose, rather than by the notions of the editor himself, as in the instance of personal proprietorship. Prompted by this belief the president declined to appoint on the publication committee the editor of the Journal. In my opinion, the salaried agents of the society ought not, for apparently obvious reasons, to be entitled to membership in the executive bodies which regulate their compensations or determine their policies of action. Other and wiser means of conference than this can easily be determined." And again: "I am clearly of the opinion that the net earnings of the Journal should be utilized for its betterment, and for the purpose of extending, when feasible, publication courtesies to such of those as contribute to its pages important and original articles. It should not be the policy of this Society, in my judgment, to accumulate worldly belongings, but instead to increase the wealth of good fellowship and professional advance, by a wise adjustment relating thereto of its business management."

INDEPENDENT PROFESSIONAL MEDICAL JOURNALS ARE MORE PECULIARLY REPRESENTATIVE OF THE PROFESSION THAN ARE SOCIETY ORGANS.—Any attack upon their existence by organized power is a direct blow at the profession itself. To them, more than any other agency, has been due the development of the movement toward solidarity, as well as the advance of science or reform. The initiative has always been theirs, and, so long as progress springs from the work of the few instead of from the work of organized bodies, they will be needed that growth may come. They alone can represent independent thought, or exercise the wholesome function of disinterested criticism. Yet under present conditions, it is in the power of a few men in the Association, by employing the competitive methods of a monopoly, to make the existence of independent journals impossible. These journals may be threatened with the boycott unless they are entirely subservient to those controlling the Association. They may be crowded out by unfair competition in the advertising field, or by pressure exerted in various ways; for example: By the Association and State journals adopting rates proportionately so low that journals unsupported by organization fees will be unable to compete; by using organization as a club, "blackmailing" the advertiser in a cunning way to secure patronage for the organization journal and bar out a rival; by the organization journals taking advertising until proved unfit, and claiming the right of such proof under control so that none may say conclusively that exclusion and ability to dispense with such revenue are coincident. Unfair competition may also exist in the matter of literary and scientific material. Efforts may be used to secure for organization journals literary and scientific material not constructively nor in any sense justly to be claimed by them. Yet these journals are frequently unable to publish fully their own transactions for "lack of space!" Authors may be shown that unless their articles are published in these organization journals they will, so far as possible, be denied quotation and reference in their pages. It is publicly stated that all these methods have been employed in concerted attack, both open and secret, upon independent medical journals of high professional standing.

TRADES-UNION METHODS are not in the spirit of the profession. They have been initiated and carried out by the few for self-aggrandisement directly, or indirectly by means of the accumulation of a large surplus. They will ultimately be condemned by the great body of the profession, but this should be done while prevention of ill-effects is still possible. It is unseemly that organization journals should carry any advertising which they may be compelled at any time to exclude or denounce. Nor should the organs of professional scientific bodies act as the mouth-piece of manufacturing or publishing firms. Higher ethics would dictate that they devote themselves to organization and professional matters, and publish no advertising. Particularly is this true if reform in advertising is to be carried out through their agency, or by their attack. General confidence in the purity of their motives will never be felt so long as present methods prevail. President Bryant speaks of these matters in no uncertain terms: "Whether or not the medical journals which, for covetous business reasons, exploit remedial agents in a manner calculated to deceive the unwary, the unsophisticated and the indolent, are to receive professional support, will, as it seems to me, be determined more by the outcome of patiently developed, and higher comprehensive professional sense, than by other inhibiting influnces."

REFORMS DEMANDED IN THE INTERESTS OF PROGRESS.—Briefly these may be summarised:

1. Verbatim reports of the proceedings of legislative and governing bodies.

2. Itemization and utmost publicity of financial matters.

3. Proper representation in the offices of Secretary, of Editor, and of Business Manager by separate individuals, with proper compensation.

4. Nondiscretionary power of the Editor, with government by the Sections of the published proceedings.

5. The rendering impossible of trades-unionism and monopolistic methods.

6. Provision for general secret ballots upon important questions of policy by means of the machinery of the Association and its Journal, through district and county societies.

7. The extension of the referendum and initiative from the optional legislative to the popular and obligatory form.

8. In order to protect apparent minorities, placing the vote necessary for both referendum or initiative upon a reasonable basis.

9. The rights of individual members must be held inviolate from attack by those in power.

10. The Association and its Journal must be enjoined from entering into purely commercial competition to the detriment of its professional rivals.

11. No paid agent of the Association should be permitted to be a member of, or take part in the deliberations of, the bodies governing or directing his actions or compensation.

These reforms are demanded by a large and increasing body of the profession, among whom are many members of the Association, and not a few of its officers. They are here presented for discussion by the House of Delegates at the approaching annual meeting. May that body have wisdom sufficient for reform from within lest reform be forced from without.

